# Community engagement and involvement in managing the COVID-19 pandemic among urban poor in low-and middle-income countries: a systematic scoping review and stakeholders mapping

**DOI:** 10.1080/16549716.2022.2133723

**Published:** 2022-12-20

**Authors:** Krushna Chandra Sahoo, Mili Roopchand Sahay, Shubhankar Dubey, Subhasish Nayak, Sapna Negi, Pranab Mahapatra, Debdutta Bhattacharya, Mariam Otmani Del Barrio, Sanghamitra Pati

**Affiliations:** a Health Technology Assessment in India (HTAIn), ICMR-Regional Medical Research Centre, Bhubaneswar, Odisha, India; b Department of Psychiatry, Kalinga Institute of Medical Sciences, Bhubaneswar, Odisha, India; c UNICEF/UNDP/World Bank/WHO Special Programme for Research and Training in Tropical Diseases (TDR), World Health Organization, Geneva, Switzerland

**Keywords:** Community engagement and involvement, urban-poor, low-and middle-income countries, COVID-19, stakeholder mapping

## Abstract

**Background:**

Community engagement and involvement (CEI) was crucial for the COVID-19 pandemic response, particularly among the urban poor in low-and middle-income countries (LMICs). However, no evidence synthesis explores how CEI can benefit public health emergencies.

**Objective:**

We conducted a systematic scoping review of the CEI with an emphasis on stakeholder identification, accountability mapping, the support system, and the engagement process among urban poor populations in LMICs during the COVID-19 pandemic.

**Methods:**

We searched eleven databases, including PubMed, Embase, Web of Science, and CINAHL, following the PRISMA-2020 guidelines to find articles published between November 2019 and August 2021. PROSPERO registration No: CRD42021283599. We performed the quality assessment using a mixed-method appraisal tool. We synthesized the findings using thematic framework analysis.

**Results:**

We identified 6490 records. After the title and abstract screening, 133 studies were selected for full-text review, and finally, we included 30 articles. Many stakeholders were involved in COVID-19 support, particularly for health care, livelihoods, and WASH infrastructure, and their accountability mapping by adopting an interest – influence matrix. This review emphasizes the significance of meaningful CEI in designing and implementing public health efforts for pandemic management among urban slum populations. The interest – influence matrix findings revealed that specific community volunteers, community-based organizations, and civil society organizations had high interest but less influence, indicating that it is necessary to recognize and engage them.

**Conclusion:**

Motivation is crucial for those with high influence but less interest, such as corporate responsibility/conscience and private food supply agencies, for the health system’s preparedness plan among urban populations.

## Background

Community engagement and involvement (CEI) are critical to achieving the Sustainable Development Goals (SDGs). CEI addresses the issues of the specific community including behavioral, cultural, and social factors, health system determinants, health prerequisites, and upstream health driving forces [1,2]. The World Health Organization (WHO) defines community engagement as establishing relationships that allow stakeholders to collaborate to address health-related issues and promote well-being to achieve good health impact and outcomes [3]. The WHO’s 13th General Programme of Work (2019–2023) aims to improve the health and well-being of the community by strengthening CEI [4]. Moreover, CEI refers to the involvement and participation of individuals, groups, and structures within the social boundary in decision-making, planning, design, governance, and service delivery [5]. The four pillars of CEI approaches are community-oriented, community-based, community managed, and community-owned [1]; all of which are crucial for the improved health status of the vulnerable population.

In 2015, 54% of the world’s population resided in urban regions is gradually increasing [6]. According to the United Nations, more than 90% of expected urban population growth would occur in low- and middle-income countries (LMICs) [7]. Hence, the CEI is crucial to understand and successfully implement various community programs such as health, livelihood, water, sanitation, and hygiene [8].

The COVID-19 pandemic has claimed countless lives and infected individuals and has had diverse effects on communities worldwide [8]. COVID-19 is more likely to infect displaced and underprivileged populations like the urban poor, where the community support system would play a vital role for disease prevention and livelihood support [5]. The public health incidents demonstrate the significance of context-appropriate CEI approaches, including community participation, for outbreak containment [5]. Recent global data have shown the importance of community health workers (CHWs) and community involvement during the COVID-19 pandemic. In the case of LMICs, however, to our knowledge, no evidence synthesis explores how CEI can benefit human public health emergencies, such as pandemics among the urban poor. Therefore, we conducted a systematic scoping review of the CEI with an emphasis on stakeholder identification, accountability mapping, the support system, and the engagement process among urban poor populations in LMICs during the COVID-19 pandemic.

## Methods

### Search strategy and selection criteria

A thorough search was conducted on eleven scholarly online repositories (PubMed/MEDLINE, Embase, Web of Science, CINAHL (EBSCO), ProQuest, Cochrane, Epistemonikos, WHO Global Index Medicus, MedRxiv and BioRxiv, 3ie Impact Evaluation Repository, and Google scholar). Primarily, we created a broad search using the terms: slums, COVID-19, LMICs (based on World Bank classification) [9], and community engagement and involvement. The inclusions and exclusions criteria were provided in Table 1.

**Table 1.** Study inclusions and exclusions criteria.

Inclusion criteria	All types of primary studies, including qualitative, quantitative and mixed-methods in the context of urban poor, community engagement involvement, COVID-19, and published between November 2019 and August 2021.
Exclusion criteria	Reviews, commentaries, editorials articles, any non-COVID-19 studies related to CEI and settings other than urban poor.

We reported this systematic review following the Preferred Reporting Items for Systematic Reviews and Meta-Analyses (PRISMA 2020) guideline [10] and the protocol is registered on PROSPERO (CRD42021283599).

All curated studies were then imported into EndNote X8 software to identify and remove duplicate records. Title and abstract screening of included articles were done by three reviewers independently using Rayyan software. We selected all primary (qualitative quantitative and mixed-method articles) peer-reviewed articles. Commentaries, editorials, perspectives, reviews, and any non-COVID-19 studies related to community engagement and involvement (CEI), non-LMICs, and settings other than slums were excluded. The full-text screening was done by two reviewers (MRS and SN) independently to ensure compliance with the study’s objectives. We excluded articles that did not include CEI data during the full-text review.

### Data extraction and synthesis

We extracted quantitative data in Microsoft Excel using a standardized template. The data included the study type, the country, the types of urban poor, the data collection method, type of stakeholder, and type of support provided. Two reviewers separately extracted data, then cross-checked and compiled it by a third reviewer.

We synthesized qualitative findings using a thematic framework analysis approach. The authors thoroughly reviewed the selected studies, and finally, developed a framework for data coding interest-influence matrix. We coded the data and extracted the key findings using MAXQDA Analytics Pro 2022. We used an interest-influence matrix for stakeholders mapping and engagement in community services among the urban poor during the COVID-19 pandemic. Influence represents a stakeholder’s power to resist, and interest indicates stakeholders’ likely concerns. Prioritizing stakeholders requires determining their interests and influence. Low-interest, low-influence stakeholders are unimportant to the program, but monitoring them may be necessary for the future. High-interest, low-influence stakeholders are more involved. Despite their interest, they have little power to change things; this group’s engagement strategy is proactive communication. Low-interest, high-influence stakeholders aren’t interested; however, this group is influential. Keep this group satisfied by holding regular meetings. The high-interest high-influence group has much interest and influence; this group’s strategy for managing stakeholders is active collaboration [11].

### Quality assessment

Quality assessment of included studies was done by two reviewers using the mixed-method-appraisal (MMAT) tool [12] and disagreement was settled by a discussion with a third reviewer.

## Results

**Figure 1.**
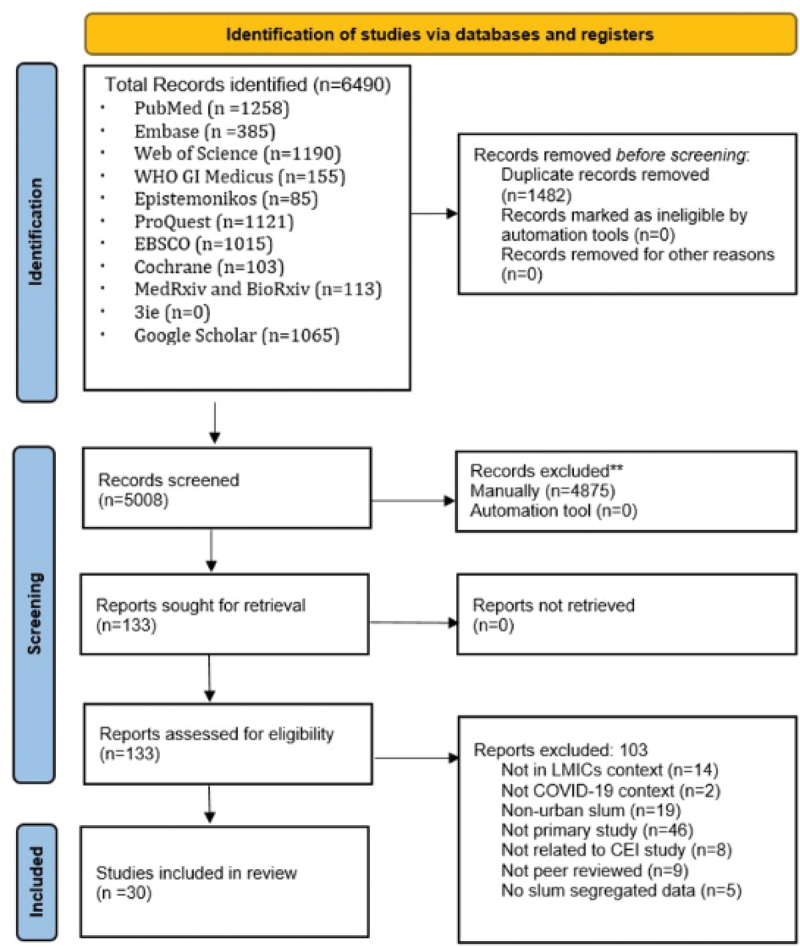
PRISMA flow diagram.

We identified 6490 records. After removing 1482 duplicates, 5008 articles were selected for the title and abstract screening. Based on inclusion criteria, we excluded 4875 studies. We reviewed the full texts of 133 studies. Finally, 30 articles included – nine quantitative, thirteen qualitative, and eight mixed-method studies. The steps involved in study selection are depicted in the PRISMA flow diagram (Figure 1). The characteristic of the included studies is provided in Table 2.

**Table 2.** Study characteristics.

Author year	Country	Study type	Study approach	Sampling	Data Collection Method	Types of urban poor	Key Stakeholder	Community engagement approach	Support domains
Baaees et al., 2021	Yemen	Quantitative	Cross-sectional	Not applicable	Surveillance	Internally displaced people	Community Volunteer	Community-based surveillance	Health
Costa et al., 2020	Brazil	Quantitative	Cross-sectional	Snowball	Survey	Slum dweller	Community Health Workers	Community health services	Health
Fransen et al., 2020	Kenya	Quantitative	Cross-sectional	Random	Survey	Slum dweller	Local NGOs	Technical and financial support	Livelihood
Hajjar and Abu-Sittah, 2021	Syrian	Quantitative	Cross-sectional	Not applicable	Survey	Refugee	Local NGOs	Care for refugee children	Health
Kar et al., 2021	India	Quantitative	Cross-sectional	Purposive	Survey	Slum dweller	City Authorities	Care taken for the special groups	Health
Kaushal and Mahajan, 2021	India	Quantitative	Cross-sectional	Not applicable	Not applicable	Slum dweller	City Authorities, Healthcare Providers, Slum-dwellers	Awareness, arrange quarantine centers and free kitchen	Health
Patel, 2020	India	Quantitative	Cross-sectional	Census	Not applicable	Slum dweller	City Authorities	Free food distribution and awareness	Livelihood
Pinchoff et al., 2021	Kenya	Quantitative	Cross-sectional	Random	Survey	Informal settlement	City Authorities, Local NGOs	soap or hand sanitizer, cash, and food.	Health
Durizzo et al., 2020	South Africa, Ghana	Quantitative	Cross-sectional	Random	Structured phone survey	Low-income settlement	City Authorities	Water and food distribution, support testing processes	Health & Livelihood
George et al., 2020	India	Mixed-method	Ethnographic	Purposive	Surveys, FGDs	Informal settlement	City Authorities, Public Health Department, Private Hospitals	Provided health services	
Nunes et al., 2021	Brazil	Mixed-method	Cross-sectional	Random	Interview	Homelessness	City Authorities	Comprehensive care and health promotion	Health
Peteet et al., 2020	India	Mixed-method	Report	Not applicable	Field Survey	Slum dwellers	Community Health Workers	Free tests for screening, health promotion, and maternal health care	Health
Pongutta et al., 2021	Thailand	Mixed-method	Cross-sectional	Not mention	Interviews	Slum dwellers	Community Volunteer	Provide food, sanitary product	Livelihood & WASH
Raman et al., 2021	India	Mixed-method	Ethnographic	Purposive	In-depth Interview	Slum dwellers	Community Volunteer	Comprehensive care and health promotion	Health
Sumalatha et al., 2021	India	Mixed-method	Cross-sectional	Random	Telephonic discussion	Slum dwellers	City Authorities	Comprehensive care and health promotion	Health
Ahmed et al., 2020	Bangladesh, Kenya, Nigeria, and Pakistan	Mixed-method	Cross-sectional	Purposive	Survey, FGDs	Slum dwellers	City Authorities, Private Hospital	Providing health services, and financial support	Health & Livelihood
Williams and Shahabuddin, 2021	Bangladesh	Mixed-method	Cross-sectional	purposive	Survey, FGDs	Slum dwellers	Community volunteers	Provide shelter and financial support	Livelihood
Akter et al., 2021	Bangladesh	Qualitative	Phenomenology	Snowball	Interview	Slum dwellers	City Authorities, Local NGOs	Water, food, and financial support	Livelihood
Bhattacharya et al., 2021	India	Qualitative	Phenomenology	Purposive	Semi-structured interview	Homeless people	Local NGO and Service Providers	Basic amenities through social media	Livelihood
Braga et al., 2020	Brazil	Qualitative	Exploratory	purposive	FGD	Informal settlement	Public Administration and Social Organization	Food kit donations, Digital food card	Livelihood
Douedari et al., 2020	Syria	Qualitative	Phenomenology	Snowball	Interviews	Displaced communities	City Authorities, NGOs, Health Facilities	Awareness campaigns, water & hygiene kits	Health
Ebekozien et al., 2021	Nigeria	Qualitative	Phenomenology	Snowball	Semi-structured interviews	Informal settlement	Government and Healthcare Providers	An alcoholic-based solution to overcome hindrance in access to water	Health
Munajed and Ekren, 2020	Lebanon and Turkey	Qualitative	Exploratory	Snowball	semi-structured interviews	Refugee	Local NGO	Social welfare aid	Livelihood
Nyashanu et al., 2020	Tshwane	Qualitative	Exploratory	Purposive	Semi-structured	Informal settlement	Local NGOs, Religious organizations	Awareness, food Aid, Washbasins	Health & WASH
Ojogiwa and Akinola, 2020	Nigeria	Qualitative	Exploratory	Purposive	Interview	Slum dwellers	City Authorities, Local NGOs	Free food and financial assistance	Livelihood
Osuteye et al. 2020	Sierra Leone	Qualitative	Not mention	Purposive	Key informant interview	Informal settlement	Community-based Organizations	Water and food distribution, support quarantine processes	Health & Livelihood
Ruszczyk et al., 2020	Bangladesh	Qualitative	Empirical	Purposive	In-depth household surveys, FGD	Informal settlement	Stakeholders	Food support	Livelihood
Srivastava et al. 2021	India	Qualitative	Phenomenology	Purposive	semi-structured interview	Migrant workers	Local NGO	Social support	Livelihood
Sudhipongpracha and Poocharoen, 2021	Kenya and Thailand	Qualitative	Phenomenology	Purposive	interview	Slum dwellers	Local NGOs and Community Health Workers	Community awareness	Health
Venkatachalam and Memon, 2020	India	Qualitative	Phenomenology	Purposive	interview	slum dwellers	Stakeholders	Free food distributions, home-based care for chronic illness	Health & Livelihood

## Stakeholder identification and accountability mapping

**Figure 2.**
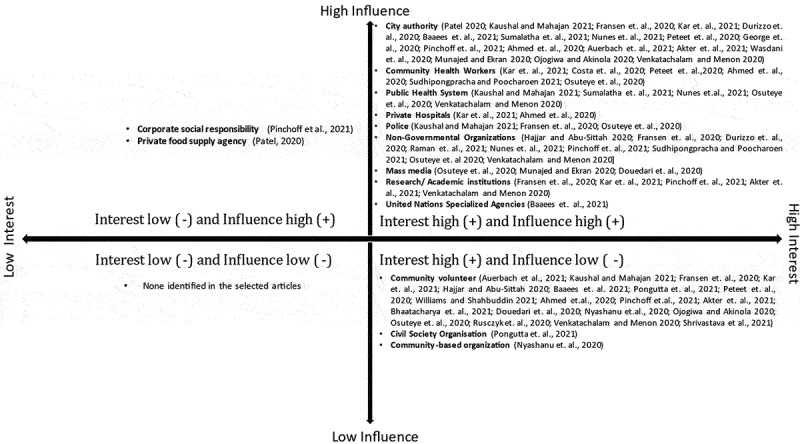
Stakeholder analysis (accountability mapping using interest–influence matrix).

Studies revealed various stakeholders with community participation had a significant role in managing COVID-19 among the urban poor of LMICs. The stakeholders were city authorities (urban local bodies), civil society groups (CSO), Community-based organizations (CBO), Community health workers (CHW), Community volunteers, corporate social responsibility (CSR) groups, mass media, local non-governmental organizations (NGOs). The police, a private food supply agency, private hospitals, the public health system, United Nations (UN) specialized organizations, researchers, and academic institutions participated in several community engagement activities and were supported differently. From the grass-root to the central level, these stakeholders supported urban slum communities on different platforms, such as awareness of the pandemic, facilitated healthcare, supply of food and water, as well as financial support. The detailed accountability mapping of various stakeholders using an interest – influence matrix is presented in Figure 2. The following symbol was used to map interest high (+), influence high (+), interest low (-), and influence low (-).

Many stakeholders were recognized to have a significant influence on the urban poor community, and they participated in community service for the urban poor during the pandemic out of personal interest. City authorities – the staff of urban local bodies, which includes both elected members (public representatives) and government administrative staffs [13–30], community health workers [16,21,24,31–33], public health functionaries [14,19,20,30,33],, police [14,15,33], research and academic institutions [15,16,23,26,30], non-governmental organizations [15,17,20,23,28,30,32–34], media [28,33,35], and United Nations special agencies [18], as well as a few private hospitals [16,24] were among these stakeholders.

‘We were given rice (60 kg), lentil (1 kilogram), salt, flour (2 kg), edible oil (1 L), sugar (1 kg), pulses, and potatoes by local government officials (2 kg). They also handed out food packages. They aided in the testing procedure in our community’ [26–28].

According to the studies, the local government extended extensive empowerment to corporate social responsibility (community development strategy of private organizations) [23] and private food supply agencies [13]. They strongly influenced community services during the pandemic, but the studies reported their lack of interest; the causes were not mentioned. On the other hand, several studies revealed that even though community volunteers [14–16,18,21,23–26,29,30,33–41], civil society organizations [36], and community-based organizations [39,42] had a high level of interest, they had the least influence – the government restricts their participation in community services, maintaining the restricted guidelines for the COVID-19 pandemic. We did not find any studies that reported on low interest and low influence stakeholders for community participation among urban poor populations during the pandemic.

‘The majority of participants said they were given soap or hand sanitizer, followed by meals. When asked what their single greatest unmet need was, the top two responses were food (94% in April, 86% in May) and cash (45% in April, 48% in May). The government, non-governmental organizations, good Samaritans/corporate sponsorship, and religious institutions all donated items’ [23].

‘Members of non-governmental organizations came to see us on a regular basis and brought us dry food and water bottles, as well as transporting a few patients to hospitals’ [38].

## Support domains and community engagement approaches

The studies identified health, livelihoods, water, sanitation, and hygiene (WASH) as significant support domains. Twelve studies (40%) described the CEI in healthcare services, such as preventative and curative services. Ten studies described CEI as a means of sustaining livelihoods during pandemics. Approximately four studies featured CEI support for health and livelihood, and one study each described health and WASH and livelihood and WASH.

### Healthcare support

For healthcare support, urban slum stakeholders organize isolation or quarantine centers, facilitate the screening and diagnosis of COVID-19, and help urban community members reach health facilities for treatment of COVID-19 infections and other non-COVID-19 related disorders.

Many studies showed that it proved challenging to maintain home isolation, quarantine, and physical distance guidelines for COVID-19 prevention among urban slum dwellers due to insufficient housing and WASH infrastructure and high population density. By transforming Anganwadi centers into COVID-19 care facilities, city officials constructed institutional quarantine for migrants [16]. About half of the slums used community places such as schools and meeting rooms to isolate themselves; nonetheless, some larger houses adopted a new furniture layout but entirely sacrificed lighting and ventilation for COVID management [26]. In Mumbai, local officials took the initiative to set up quarantine facilities in nearby schools, sports complexes, marriage palaces, and community halls to provide critical care in collaboration with private hospitals and non-governmental organizations [14].

In many countries, the city officials, medical professionals from government hospitals, community health workers, and community volunteers were engaged in surveillance, contact tracing, and hygiene and preventative awareness initiatives related to COVID-19 [16,18,31]. Community action groups in Malvani assisted municipal employees in addressing the difficulty of tracking and detecting positive COVID-19 cases in densely inhabited slums. To help restrict the spread of infection, government officials tattooed the palms of affected people with the help of local community volunteers [22,30]. Community action groups assisted local NGOs in developing COVID-19 awareness, control, and prevention messaging in local languages that were simple to grasp, contextualized, and acceptable to the communities [30]. The community health workers raised community awareness about positive cases and guidelines. They also advised slum dwellers to avoid congested routes or places [14,21]. Community health professionals began raising community awareness in Thailand and Kenya, but the primary challenges were convincing people to collaborate and probable violence from some community members [32].

The health facility focal points and community health workers enabled referrals for people with flu-like symptoms and positive cases, either home care or admission to healthcare facilities [18,21]. Patients were referred to government hospitals, community clinics, and mobile clinics, and medicine was given to them [21]. Many non-governmental organizations (NGOs) in Bangladesh offer emergency care [24]. Community health professionals in Kenya, Nigeria, and Pakistan provided screening services through fixed/mobile primary healthcare clinics [24]. Community health workers in India recognized cases and followed up on symptomatic patients, in addition to other routine health programs such as Urban Health and Nutrition Day and Routine Immunization Day [16].

### Livelihood support

Economic difficulties during COVID-19 contributed to the food insecurity [19,26] and poor quality of life [28]. About sixty percent of households chose to cut back on food, while sixty-five percent skipped meals. Approximately 5% sold home items to finance food-related expenses. Many relied on minimal aid from non-governmental organizations [19,26]. Furthermore, closing public schools that provided free lunches, loss of income, and rising food prices were obstacles for joint families [27]. They believed they would die of hunger rather than the virus, so they borrowed money from relatives or neighbors to purchase milk and bread for their children rather than masks, gloves, and expensive sanitizer [27]. Few urban women established modest businesses, such as tiffin booths, relying on government subsidies to support their families [16,19].

The homeless and street dwellers hoarded the food donated by local NGOs and handed it to them [38,42]. Numerous family members borrowed money to meet their fundamental wants [19,26,28]. Many urban poor relied on money to endure the lockout [26,27].

### WASH support

The lockdown regulations have stressed the strained mobile toilets (often known as ‘duped chemical toilets’) [39]. The number of portable toilets has been increased to fifty to serve a larger population. People use nearby bushes as toilets [39]. The slum community was promptly supplied with piped water, making it easier to wash hands frequently and maintain a clean home. Using sanitizers and masks provided by the government [28,43], urban residents could engage in cleanliness activities. At various times, 91% of slum residents utilized soap and water for hygiene routines supported by NGOs [36,39]. During the same activities, those who did not use soap opted to wash their hands with running water [16]. Due to the paucity of toilets, baths, and water sources, only 10% could afford to construct improvised toilets by borrowing from family and friends without NGO or government assistance/loans/subsidies [26].

Many urban poor populations could not buy gloves, masks, and disinfectants and had limited access to water, making it impossible for them to maintain COVID preventative measures [26]. Others utilized homemade masks or received free masks and soaps from non-governmental organizations due to the lack of affordability [26]. Nonetheless, 95% of residents stopped using the masks and soaps after NGOs ceased providing them [26]. Most relied on government and non-government organizations for sanitizers and masks to overcome this condition [28,43]. They also chose an alcohol-based hand-washing liquid [26].

## Discussion

This study systematically reviews community engagement and involvement (CEI) in managing the COVID-19 pandemic among the urban poor in LMICs. It defines the many stakeholders involved in COVID-19 support, particularly for health care, livelihoods, and WASH infrastructure, as well as their accountability mapping by adopting an interest – influence matrix [high interest (+), strong influence (+), low interest (-), and low influence (-)]. Typically, community engagement entails spending time with communities to establish trust and guarantee that community representation structures are inclusive and responsible. The CEI entailed the participation of public and private stakeholders, from the local to the international level, primarily the local community volunteers, to support the community during any humanitarian disaster. The evidence suggests that motivation is an important determinant of effective CEI.

Past pandemic responses to Ebola outbreaks have highlighted the significance of community engagement for the successful long-term management of infectious disease epidemics [44,45]. Experience from the 2014–2015 Ebola outbreak and other epidemics has highlighted the crucial role of community leaders as conduits for effective communication and meaningful community engagement in infection identification and control efforts [46]. The value of understanding local customs, beliefs, knowledge, and practices and the necessity of including meaningful community engagement with proven disease control techniques in disease prevention and control initiatives.

Community engagement for health is the process of establishing relationships that allow members of a community and organizations to collaborate to address health-related issues and promote well-being to produce good health impacts and outcomes [1]. The community is not a passive player in community engagement for health; instead, it plays an active role in addressing and resolving health challenges [2–4]. The COVID-19 pandemic has significantly influenced the health, lives, and livelihoods of individuals and communities worldwide, especially the most vulnerable population [47]. Even in nations where the pandemic has been successfully managed, communities that are underprivileged, marginalized, and alienated from most of the population remain at a higher risk due to their low income, poor living conditions, or lack of access to health care [46]. Community participation helps to maximize the effectiveness of COVID-19 readiness, response, and recovery activities at the community level to prevent and contain transmission [4]. Community engagement can also help the health sector prepare for and respond to the needs and problems of various communities in contextually appropriate ways, as well as address health and gender disparities during and after the pandemic. As a result, community governance institutions should be strengthened to leverage existing processes and create capacity among national and local stakeholders.

Community engagement is critical during an emergency and crucial for ensuring culturally relevant responses [5,48]. Populations at risk may not have enough access to government and social services, especially during health crises. They are frequently excluded from the national, subnational, and local response and relief programs. Additionally, these vulnerable populations may have had harmful interactions with health and other authorities, resulting in a lack of trust in institutions. In addition, many disadvantaged groups are not captured by existing institutional frameworks [1,4]. This review highlighted the importance of active engagement of community health professionals in surveillance and data gathering to enhance the community-based participatory techniques [1,4,5,48]. The expertise of recognized community organizations is required in order to support vulnerable populations, maintain the delivery of major services, provide critical resources to engage and empower them. Regional and municipal administrations must undertake infection prevention measures and provide essential public services. As COVID-19 grows, more families, especially urban poor, have trouble meeting basic health needs. During COVID-19, the government considers expanding community-level case-management services and innovative methods to provide social support for vulnerable populations. Reduce barriers for slum and homeless isolation in community centres and schools.

Community engagement facilitates the formation of social dynamics based on power and control that perpetuate the marginalization of groups. The legitimacy of the actors involved in mobilization and decision-making must be acknowledged by the rest of the community [48]. It is of the utmost importance to comprehend and support the inventiveness of community partners and key stakeholders as they devise means to engage with their people during the pandemic. In addition to leadership, buy-in for many community activities, maintaining a balance between power and the representation of many viewpoints is also essential [1,4,5,48].

The most significant reason for identifying and comprehending stakeholders is that it allows for their active participation in community services. The key stakeholders, according to this review, are usually government officials and policymakers such as legislators, mayors, city/town councilors, and local NGOs. However, this review portrayed that during an emergency or in the case of any community development initiative, the media and community leaders are the major motivators and liaising groups among community members, in this case, the urban poor population, and government agencies. Community stakeholders such as neighborhoods, community development groups, development organizations, and NGOs frequently serve as immediate service providers for emergency preparedness and key motivators for community awareness on adherence to emergency guidelines [15–35]. Hence, facilitating highly engaged stakeholder participation in discussions is critical [16,21,24,31–33]. Taking these actions before and during participant involvement will result in better stakeholder engagement and outcomes. Stakeholders often participate based on their interest in the community development program and ability to contribute; however, their involvement will also be determined by the extent to which the program outcomes will influence those being consulted [31–34]. Therefore, there is a urge for identification of motivational factors to ensure active and sustainable CEI during any emergency.

The first step toward a successful implementation of a CEI programme is to conduct an analysis of the various stakeholders. It is preferable that individuals, as well as private or public organizations and individuals or groups, who are involved in community development and service could be considered stakeholders [49]. According to an interest – influence matrix, the influence, and interests of diverse stakeholders are crucial for delivering effective services. Moreover, the motivation is key to continued stakeholder engagement with CEI. Ninety percent of the COVID-19 pandemic cases have been recorded in urban areas. Cities are bearing the brunt of the problem, with many suffering from overburdened health systems, insufficient water and sanitation facilities, and other difficulties [50]. The individuals living in slums have challenges in terms of livelihood, WASH, housing, and health care. Because of their living conditions – overcrowding, substandard housing, and shared toilets and water points – slums and informal settlements are not prepared to deal with the pandemic. Therefore, in order to combat the pandemic for the urban poor frequently encourage better CEI. This review showed that city officials, community health workers, police, the public health system, local and international NGOs, and the mass media all played critical roles in the prevention and rehabilitation of the urban poor population during pandemic; they were the highly influential and interested stakeholders [13–30]. Therefore, it is recommended to closely manage, engage, and motivate the above stakeholders for the purpose of the CEI sustainability for health system preparedness among urban poor. The local government also urged the CSR group and the private food agency to actively participate in CEI during the pandemic [13,23]. Even though this group is powerful or has a great deal of influence, they have little interest; consequently, for any future emergency preparedness plan, it will be required to maintain their satisfaction by holding regular meetings through orientation and awareness. On the other hand, although community volunteers, community-based organizations, and civil society platforms have a strong desire to serve the community, they have received little assistance or permission from the government [14–16,18,21,23–26,29,30,33–42]. Consequently, we may keep these parties in the loop, informing, engaging, and equipping them appropriately for involvement. We must adequately monitor the less influential and less invested parties [51].

This systematic review is one of its kind to entail the role of CEI for the urban poor using influence -interest matrix during a pandemic. It advocates strengthening of CEI to avert any crisis among the urban poor during pandemic or any other public health emergencies. This review is methodologically rigorous; however, very limited studies define the clear role definition of each stakeholder. Moreover, the findings from this review are limited to the urban poor residing in LMICs only, as the studies on urban poor from higher-income countries were not considered.

## Conclusion

This review emphasizes the significance of meaningful participation and engagement of diverse stakeholders in designing and implementing public health efforts for pandemic management among urban slum populations. The interest – influence matrix findings revealed that specific community volunteers, community-based organizations, and civil society organizations had high interest but less influence, indicating that it is necessary to recognize, engage, and empower them. Similarly, motivation is crucial for those with high influence but less interest, such as corporate social responsibility and private food supply agencies, for the health system’s preparedness plan among urban populations.

## Data Availability

Data will be made available on request.

## References

[1] Community engagement: a health promotion guide for universal health coverage in the hands of the people [Internet]. [cited 2022 Sep 18]. Available from: https://www.who.int/publications-detail-redirect/9789240010529

[2] Questa K, Das M, King R, Everitt M, Rassi C, Cartwright C. Community engagement interventions for communicable disease control in low-and lower-middle-income countries: evidence from a review of systematic reviews. Int J Equity Health. 2020 Dec;19:1–12. 10.1186/s12939-020-01169-5 PMC7137248 32252778

[3] WHO community engagement framework for quality, people-centred and resilient health services [Internet]. [cited 2022 Sep 18]. Available from: https://www.who.int/publications-detail-redirect/WHO-HIS-SDS-2017.15

[4] Role of community engagement in situations of extensive community transmission of COVID-19 [Internet]. [cited 2022 Sep 18]. Available from: https://www.who.int/publications-detail-redirect/WPR-DSE-2020-016

[5] Gilmore B, Ndejjo R, Tchetchia A, Claro V, Mago E, Lopes C, et al. Community engagement for COVID-19 prevention and control: a rapid evidence synthesis. BMJ Glob Health. 2020 Oct 1;5:e003188. 10.1136/bmjgh-2020-003188 PMC7554411 33051285

[6] World’s population increasingly urban with more than half living in urban areas | UN DESA | United Nations department of economic and social affairs [Internet]. [cited 2022 Sep 18]. Available from: https://www.un.org/en/development/desa/news/population/world-urbanization-prospects-2014.html

[7] Bolay JC. When inclusion means smart city: Urban planning against poverty. In: Proceedings of the Future Technologies Conference. 2019 Oct 24. Cham: Springer; p. 283–299.

[8] Ritchie D, Parry O, Gnich W, Platt S. Issues of participation, ownership and empowerment in a community development programme: tackling smoking in a low-income area in Scotland. Health Promot Int. 2004 Mar 1;19:51–59. 14976172 10.1093/heapro/dah107

[9] World Bank Country and Lending Groups – World Bank Data Help Desk [Internet]. [cited 2022 Sep 18]. Available from: https://datahelpdesk.worldbank.org/knowledgebase/articles/906519-world-bank-country-and-lending-groups

[10] Page MJ, McKenzie JE, Bossuyt PM, Boutron I, Hoffmann TC, Mulrow CD. The PRISMA 2020 statement: an updated guideline for reporting systematic reviews. BMJ. 2021;372:n71. 33782057 10.1136/bmj.n71 PMC8005924

[11] Hearn S Alignment, interest, influence matrix. [cited 2014 Sep 15]. Available from: https://www.outcomemapping.ca/nuggets/alignment-interest-influence-matrix

[12] Hong QN, Pluye P, Fàbregues S, Bartlett G, Boardman F, Cargo M, et al. Mixed methods appraisal tool (MMAT), version 2018. Regist Copyr J. 2018 Aug;34(4):285-91.

[13] Patel A. Preventing COVID‐19 amid public health and urban planning failures in slums of Indian cities. World Med Health Policy. 2020 Sep;12:266–273. 32837773 10.1002/wmh3.351 PMC7404953

[14] Kaushal J, Mahajan P. Asia’s largest urban slum-Dharavi: a global model for management of COVID-19. Cities. 2021;111:1–7. 10.1016/j.cities.2020.103097 PMC7832248 33519012

[15] Fransen J, Peralta DO, Vanelli F, Edelenbos J, Olvera BC. The emergence of urban community resilience initiatives during the COVID-19 pandemic: an international exploratory study. Eur J Dev Res. 2022 Feb;34:432–454. 33456209 10.1057/s41287-020-00348-y PMC7802407

[16] Kar S, Mohapatra I, Mishra A, Banerjee A. Mitigation strategies and Covid appropriate and risk behavior: a descriptive study at slums of Bhubaneswar. Odisha Adv Res J Multidisc Disco. 2021 May 20;57:01–6.

[17] Durizzo K, Asiedu E, Van der Merwe A, Van Niekerk A, Günther I. Managing the COVID-19 pandemic in poor urban neighborhoods: the case of Accra and Johannesburg. World Dev. 2021 Jan;137:105175. 32904458 10.1016/j.worlddev.2020.105175 PMC7455159

[18] Baaees MS, Naiene JD, Al-Waleedi AA, Bin-Azoon NS, Khan MF, Mahmoud N, et al. Community-based surveillance in internally displaced people’s camps and urban settings during a complex emergency in Yemen in 2020. Confl Health. 2021 Dec;15:1–5 34225760 10.1186/s13031-021-00394-1 PMC8256204

[19] Sumalatha BS, Bhat LD, Chitra KP. Impact of covid-19 on informal sector: a study of women domestic workers in India. Indian Econ J. 2021 Sep;69:441–461.

[20] Nunes NR, Rodriguez A, Cinacchi GB. Health and social care Inequalities: the impact of COVID-19 on people experiencing homelessness in Brazil. Int J Environ Res Public Health. 2021 Jan;18:5545. 34067316 10.3390/ijerph18115545 PMC8196886

[21] JO P, Hempton L, Peteet M JR K. Asha’s response to COVID-19: providing care to slum communities in India. Christ J Glob Health. 2020 Nov 9;7:52–57.

[22] George CE, Inbaraj LR, Rajukutty S, Witte LP. Challenges, experience and coping of health professionals in delivering healthcare in an urban slum in India during the first 40 days of COVID-19 crisis: a mixed method study. BMJ Open. 2020;10:e042171. v 1: DOI:10.1136/bmjopen-2020-042171. PMC7677374 33208338

[23] Pinchoff J, Austrian K, Rajshekhar N, Abuya T, Kangwana B, Ochako R. Gendered economic, social and health effects of the COVID-19 pandemic and mitigation policies in Kenya: evidence from a prospective cohort survey in Nairobi informal settlements. BMJ Open. 2021 Mar 1;11:e042749. DOI:10.1136/bmjopen-2020-042749 PMC7931215 33658260

[24] Ahmed SA, Ajisola M, Azeem K, Bakibinga P, Chen YF, Choudhury NN. Impact of the societal response to COVID-19 on access to healthcare for non-COVID-19 health issues in slum communities of Bangladesh, Kenya, Nigeria and Pakistan: results of pre-COVID and COVID-19 lockdown stakeholder engagements. BMJ Glob Health. 2020 Aug 1;5:e003042. 10.1136/bmjgh-2020-003042 PMC7443197 32819917

[25] Auerbach AM, Thachil T. How does Covid-19 affect urban slums? Evidence from settlement leaders in India. World Dev. 2021;140:105304. 34580560 10.1016/j.worlddev.2020.105304 PMC8457827

[26] Akter S, Hakim SS, Rahman MS. Planning for pandemic resilience: COVID-19 experience from urban slums in Khulna. Bangladesh J Urban Manag. 2021 Dec 1;10:325–344.

[27] Wasdani KP, Prasad A. The impossibility of social distancing among the urban poor: the case of an Indian slum in the times of COVID-19. Local Environ. 2020 May 3;25:414–418.

[28] Munajed DA, Ekren E. Exploring the impact of multidimensional refugee vulnerability on distancing as a protective measure against COVID-19: the case of Syrian refugees in Lebanon and Turkey. J Migr Health. 2020;1–2:100023. 10.1016/j.jmh.2020.100023 PMC8352139 34405174

[29] Ojogiwa OT, Akinola A. The impact of government responses to COVID-19 on the Urban poor in Lagos State, Nigeria. Afr J Gov Dev. 2020 Oct;1:367–381.

[30] Venkatachalam P, Memon N Community Engagement to Tackle COVID-19 in the Slums of Mumbai [Internet]. [cited 2022 Sep 18]. Available from: https://www.bridgespan.org/insights/library/global-development/community-engagement-tackle-covid-19-mumbai-slums

[31] Do Rosário Costa N, Bellas H, da Silva PRF, de Carvalho PVR, Uhr D, Vieira C, et al. Community health workers’ attitudes, practices and perceptions towards the COVID-19 pandemic in Brazilian low-income communities. Work Read Mass. 2021;68:3–11. DOI:10.3233/WOR-205000 33427724

[32] Sudhipongpracha T, Poocharoen OO. Community Health Workers as Street-level Quasi-Bureaucrats in the COVID-19 Pandemic: the Cases of Kenya and Thailand. J Comp Policy Anal Res Pract [Internet]. 2021 Mar 4. [cited 2022 Sep 18]:23:234–249. DOI:10.1080/13876988.2021.1879599.

[33] Osuteye E, Koroma B, Macarthy JM, Kamara SF, Conteh A. Fighting COVID-19 in Freetown, Sierra Leone: the critical role of community organisations in a growing pandemic. Open Health [Internet]. 2020 Jan 1;1:51–63. DOI:10.1515/openhe-2020-0005.

[34] Hajjar MS, Abu-Sittah GS. The multidimensional burden of COVID-19 on Syrian refugees in Lebanon. J Glob Health. 2021 Jan 16;11:05003. 33643636 10.7189/jogh.11.05003 PMC7897425

[35] Napier-Raman S, Rattani A, Qaiyum Y, Bose V, Seth R, Raman S. Impact of COVID-19 on the lives of vulnerable young people in New Delhi, India: a mixed method study. BMJ Paediatr Open. 2021 Jul 26;5:e001171. DOI:10.1136/bmjpo-2021-001171. PMID: 34345717; PMCID: PMC8316697. PMC8316697 34345717

[36] Pongutta S, Kantamaturapoj K, Phakdeesettakun K, Phonsuk P. The social impact of the COVID-19 outbreak on urban slums and the response of civil society organisations: a case study in Bangkok, Thailand. Heliyon [Internet]. 2021 May 1;7:e07161. DOI:10.1016/j.heliyon.2021.e07161. 34136704 PMC8180617

[37] Williams S, Shahabuddin SJ Concerns, changes and challenges faced by the extreme urban poor in Dhaka and Chittagong during the COVID-19 lockdown: June follow-up survey [Internet]. 2020. Available from: https://www.semanticscholar.org/paper/Concerns%2C-changes-and-challenges-faced-by-theurban/8abe2d70329b4c26c875262d41476915ecf8030b

[38] Bhattacharya P, Khemka GC, Roy L, Roy SD. Social injustice in the neoliberal pandemic era for homeless persons with mental illness: a qualitative inquiry from India. Front Psychiatry. 2021 Jun;12. [Internet]. DOI:10.3389/fpsyt.2021.635715 PMC8245767 34220566

[39] Nyashanu M, Simbanegavi P, Gibson L. Exploring the impact of COVID-19 pandemic lockdown on informal settlements in Tshwane Gauteng Province, South Africa. Glob Public Health [Internet]. 2020;15:1443–1453. DOI:10.1080/17441692.2020.1805787 32780633

[40] Ruszczyk HA, Rahman MF, Bracken LJ, Sudha S. Contextualizing the COVID-19 pandemic’s impact on food security in two small cities in Bangladesh. Environ Urban . 2021 Apr;33:239–254. DOI:10.1177/0956247820965156. 34253941 PMC8261459

[41] Srivastava A, Arya YK, Joshi S, Singh T, Kaur H, Chauhan H. Major stressors and coping strategies of internal migrant workers during the COVID-19 pandemic: a qualitative exploration. Front Psychol. 2021;12. [Internet]. DOI:10.3389/fpsyg.2021.648334. PMC8173127 34093333

[42] Braga MF, Romeiro Filho E, Mendonça RM, Oliveira LG, Pereira HG. Design for Resilience: mapping the needs of Brazilian communities to tackle COVID-19 challenges. Strateg Des Res J. 2020 Dec 23;13:374–386.

[43] Ebekozien A, Aigbavboa C, Ayo-Odifiri SO. Root cause of factors enhancing the spread of coronavirus disease 2019 pandemic in Nigerian informal urban settlements: issues and possible solutions. Int Plan Stud [Internet]. 2022 Jan 2;27:44–61. DOI:10.1080/13563475.2021.1917342.

[44] Kickbusch I, Reddy KS. Community matters–why outbreak responses need to integrate health promotion. Glob Health Promot. 2016 Mar;23:75–78. [Internet]. DOI:10.1177/1757975915606833. 26518038

[45] Laverack G, Manoncourt E. Key experiences of community engagement and social mobilization in the Ebola response. Glob Health Promot. 2016 Mar;23:79–82. [Internet]. DOI:10.1177/1757975915606674. 26518037

[46] Marais F, Minkler M, Gibson N, Mwau B, Mehtar S, Ogunsola F, et al. A community-engaged infection prevention and control approach to Ebola. Health Promot Int [Internet]. 2015 Feb 12;31:440–449. Available from. 10.1093/heapro/dav003. 25680362

[47] Nutbeam D. The vital role of meaningful community engagement in responding to the COVID-19 pandemic. Public Health Res Pr [Internet]. 2021 Mar;10. DOI:10.17061/phrp3112101 33690782

[48] Marsh EE, Kappelman MD, Kost RG, Mudd-Martin G, Shannon J, Stark LA, et al. Community engagement during COVID: a field report from seven CTSAs. J Clin Transl Sci [Internet]. 2021;5. DOI:10.1017/cts.2021.785 PMC8185415 34192058

[49] Brugha R, Varvasovszky Z. Stakeholder analysis: a review. Health Policy Plan [Internet]. 2000 1;15:239–246. DOI:10.1093/heapol/15.3.239 11012397

[50] Friesen J, Pelz PF. COVID-19 and slums: a pandemic highlights gaps in knowledge about urban poverty. JMIR Public Health Surveill. 2020 Sep 4;6:e19578. DOI:10.2196/19578 32877347 PMC7486000

[51] Thompson R. Stakeholder analysis. Mind Tools [Internet]. 2012;7. Available from http://ncwwi.org/files/LAMM/eLearning_files/stakeholderanalysis.pdf

